# Cell Surface Binding and Lipid Interactions behind Chemotherapy-Drug-Induced Ion Pore Formation in Membranes

**DOI:** 10.3390/membranes11070501

**Published:** 2021-06-30

**Authors:** Md. Ashrafuzzaman, Zahid Khan, Ashwaq Alqarni, Mohammad Alanazi, Mohammad Shahabul Alam

**Affiliations:** 1 Department of Biochemistry, College of Science, King Saud University, Riyadh 11451, Saudi Arabia; zahidatkhan@yahoo.com (Z.K.); ashwaqabdullah.92@gmail.com (A.A.); msanazi@ksu.edu.sa (M.A.); 2 Department of Chemical Engineering, College of Engineering, King Saud University, Riyadh 11451, Saudi Arabia; ashrafmd@gmail.com; 3 Department of Engineering Sciences, Applied Materials Science, Uppsala University, 75103 Uppsala, Sweden

**Keywords:** chemotherapy drug, cell surface, liposome, membrane, ion pore, atomic force microscopy, numerical computation, screened Coulomb interaction

## Abstract

Chemotherapy drugs (CDs) disrupt the lipid membrane’s insulation properties by inducing stable ion pores across bilayer membranes. The underlying molecular mechanisms behind pore formation have been revealed in this study using several methods that confirm molecular interactions and detect associated energetics of drugs on the cell surface in general and in lipid bilayers in particular. Liposome adsorption and cell surface binding of CD colchicine has been demonstrated experimentally. Buffer dissolved CDs were considerably adsorbed in the incubated phospholipid liposomes, measured using the patented ‘direct detection method’. The drug adsorption process is regulated by the membrane environment, demonstrated in cholesterol-containing liposomes. We then detailed the phenomenology and energetics of the low nanoscale dimension cell surface (membrane) drug distribution, using atomic force microscopy (AFM) imaging what addresses the surface morphology and measures adhesion force (reducible to adhesive energy). Liposome adsorption and cell surface binding data helped model the cell surface drug distribution. The underlying molecular interactions behind surface binding energetics of drugs have been addressed in silico numerical computations (NCs) utilizing the screened Coulomb interactions among charges in a drug–drug/lipid cluster. Molecular dynamics (MD) simulations of the CD-lipid complexes detected primarily important CD-lipid electrostatic and van der Waals (vdW) interaction energies. From the energetics point of view, both liposome and cell surface membrane adsorption of drugs are therefore obvious findings. Colchicine treated cell surface AFM images provide a few important phenomenological conclusions, such as drugs bind generally with the cell surface, bind independently as well as in clusters of various sizes in random cell surface locations. The related adhesion energy decreases with increasing drug cluster size before saturating for larger clusters. MD simulation detected electrostatic and vdW and NC-derived charge-based interactions explain molecularly of the cause of cell surface binding of drugs. The membrane binding/association of drugs may help create drug–lipid complexes with specific energetics and statistically lead to the creation of ion channels. We reveal here crucial molecular understanding and features of the pore formation inside lipid membranes that may be applied universally for most of the pore-forming existing agents and novel candidate drugs.

## 1. Introduction

Chemotherapy drugs (CDs) were recently found to induce ion pores inside lipid bilayer membranes [1,2]. These pores resemble some of the peptide-induced ion channels [3,4,5,6,7]. Peptide ion channels are formed due to quantitative adsorption of peptides in the hydrophobic regions spanning both monolayers of lipid membranes. These channels are energetically stabilized inside lipid bilayers due to specific peptide–lipid molecular interactions, confirmed theoretically [8] and in molecular dynamics (MD) simulations [9]. Although CDs (colchicine (Col) and taxol (TXL)) are known to induce ion pores across planar lipid bilayers [1,2], none has so far been investigated regarding the statistical nature of the agents’ membrane adsorption and physical energetics of the agents’ membrane binding. Moreover, we have no clue how CDs might get distributed on the cell surface before their expected accommodation (due to pore forming potency) inside the membrane through localized reorganization alongside oriented lipids. The statistical nature of the cell surface distribution, the energetics of the cell surface adsorption, etc. may play crucial roles behind the pore formation of drugs inside cell membranes. The biophysical understanding of these molecular mechanisms has been especially elucidated in this study considering the effects of the important drug candidate Col, which is chosen as an example agent.

To test the quantitative adsorption of CDs in lipid bilayers, a precondition for the formation of ion pores in lipid bilayers [1,2], we decided here to first investigate the liposome adsorption of CDs using our recently patented technology ‘Direct Detection Method (DDM)’ [10]. DDM helps us quantify directly the relative mole fraction of CDs bound to the liposomes being incubated with drugs in aqueous phase. DDM detects a substantial proportion of CDs inside liposomes. The CD adsorption is modestly perturbed due to the presence of bilayer environment modulating cholesterol in the liposome.

The quantitative membrane adsorption of CDs (confirmed in liposome systems) and membrane induction of specific CD pores (confirmed in planar lipid bilayers) led us to investigate further towards understanding the physical state of the distribution of drugs being adsorbed on the cell surface. Both cell surface adsorption and distribution of drugs are predicted to maintain specific physical energetics following statistical mechanical formula. The geometrical nature of the drug distribution might elucidate the reasons behind creation of ion pore type events on the cell surface/membrane that are observed to get induced due to certain drugs, including CDs. Taking Col as an example drug that is known to induce ion pores in model membrane system, we planned a set of experiments to address here the possible cell surface binding of the membrane-active agent (MAA). atomic force microscopy (AFM) is a cantilever imaging technique that can address general cell surface profiles. Surface adsorbed nanoparticles or biomolecules are monitored utilizing AFM techniques. We measure the shifts in heights on various locations of the surface [11,12,13,14,15]. AFM images may thus demonstrate cell surface geometry, which can be processed to measuring the surface bumps happening at low nanometer (nm) resolutions. AFM technique is also helpful to measure the adhesion force. The adhesion force can be used to calculate the adhesion energy of nanoparticles on the cell surface and thus we understand certain surface profiles of the distribution. AFM techniques were therefore chosen in this study with a view of addressing the statistical mechanics of the cell surface adsorption process of CD molecules. In summary, two important aspects were primarily addressed based on AFM data, namely: (i) inspecting the change of cell surface geometrical profile or surface roughness, (ii) and measuring the cell surface adhesion forces.

Living cells are observed to react to alterations such as cell spreading, structural perturbations of membranes and nuclei [16,17]. AFM is a versatile tool [11] used in biological science [11,12,13,14,15,16,17,18]. It is capable of resolving molecular details of the surface of the cell under ambient conditions [19,20]. AFM may address the low nm scale geometrical organization and dynamics of cell membranes and cell walls, measuring cell mechanics and cell adhesion [21]. Possible CD adsorption on the cell surface, the nm scale organization, can therefore be easily inspected using AFMs.

General agents or specific drugs predicted to bind or interact with cell membrane-based targets must undergo considerable cell surface adsorption in a mainly hydrophilic liquid environment. They require to ensure their reasonable statistical presence at the hydrophilic/hydrophobic cell membrane boundary before heading towards pure hydrophobic cell membrane core regions. Phenomenological address of this drug migration leading to, or being caused by, certain molecular mechanisms resulting any specific cell membrane actions—such as gross membrane permeabilization via creating stable ion pores or instantaneous defects, etc.—is crucial. Electrophysiological experiments recorded ion currents across any lipid membranes, doped with various membrane proteins (MPs), antimicrobial peptides (AMPs), etc., may help investigate the possibility of constructing any protein/peptide–lipid complexes leading to the creation of any ion pores/channels [3,4,5,6,7,22,23,24]. Using this electrophysiology technique, we already found two premier CD agents [25,26,27,28,29] to induce events inside membrane analogous to AMP-induced ion channels [1,2]. Molecular mechanisms of drug effects on cell membranes were earlier elucidated by performing in silico MD simulations of various CD–lipid complexes [2]. A thorough analytical analysis on physical diffusion of the cell-active agents (CAAs) considering their geometrical localizations across membranes and other inter- and intra-structural cell boundaries has recently been made (see [30,31]). Various parameters related to the statistical distribution of CAAs have been inspected via measuring CAAs’ mobility. All these various theoretical and experimental techniques altogether help us understand the mechanisms of general actions and statistical nature of the distribution of CAAs and MAAs (e.g., specifically CDs) on or across cell surface regions, but often indirectly. A direct measurement using some valid scientific technique is therefore due. DDM and AFM appear as two strong techniques capable of measuring/directly inspecting the adsorbed CDs inside liposomes and on cell surfaces, respectively.

CD candidates, Col and taxol, are widely known to act upon cell generally and cell membrane specifically [32,33,34,35,36,37,38]. Our recent addition to this knowledge was that both Col and taxol induce ion conduction pores across cell membranes. Supplementary Figure S1 shows the recorded representative membrane currents across lipid bilayers. Detailed inspections of the current traces demonstrate possible ‘toroidal type’ ion pore induction by CDs (for details see our previous publications [1,2]). Due to all these known effects of CDs on membrane’s electrical and physical properties [1,2,35,36,37,38,39,40,41], we thought it might be found important from especially drugs’ off-target binding (causing gross cytotoxicity) point of view to address their specific lipid membrane adsorption as well as the general cell surface binding phenomena. DDM addresses the liposome adsorption and AFM imaging addresses the cell surface binding of drugs (Col was chosen for both studies as an example CD candidate). In the liposome binding assays, we have quantified the bilayer adsorption of Col. We also found that the quantitative bilayer adsorption is modulated due to the presence of other biochemicals, e.g., cholesterol (tested here) in the lipid bilayer. In cell assays, we have inspected the statistical nature of the cell surface distribution of CD agents that leads to modeling the drug distribution on the cell surface and raising our understanding of the underlying energetics of membrane adsorption of drugs. We have achieved three specific fundamental objectives in this cell surface research:

(i) address the statistical significance of the cell surface binding of CDs;
(ii) understand the nature of the surface distribution (random or cluster creation) of drug molecules and propose drug distribution models; and
(iii) finally, measure the energetics of cell surface adsorption of drugs.

Following the experiments, we performed a set of numerical computations (NCs) and measured the free energy of drug–drug and drug–lipid association in a drug cluster and lipid cluster, respectively, in lipid membranes. The AFM detected clusters of drugs and drug–lipid complexes on the cell surface help model the cell surface distribution of drugs, create templates for NCs to theoretically address the energetics of drug adsorption on the surface of the cell. As phosphatidylcholine (PC) is the major lipid in the cell membrane, we considered a cluster of PCs (PC domain) to inspect the Col interactions with PCs. Here we have derived analytical energy expression in Fourier space using screened Coulomb interaction (SCI) formalism, identical to the energy expression we developed recently for understanding the free energy of association for gramicidin A (gA) channel in a lipid membrane (for details, see [8,24]). The detected energies and associated parameters help us compare the CD effects directly with other ion channel forming peptide effects on model membrane systems, theoretically. Thus, the molecular understanding on the energetics behind CD-induced ion pore formation may get elucidated in NCs.

The NC detected energies presented in this article importantly hint that the charge-based SCI interactions among drugs and lipids are behind drug binding with the cell membrane lipids and that these interactions possibly lead to the creation of the drug clusters detected in AFM imaging [11,12,13,14,15,42,43]. These SCI interactions and clustering of agents/drugs are both fast physical phenomena. Of course, as a precondition a rather slower and mainly biochemical phenomenon, the cell surface/membrane adsorption of drugs in the hydrophilic/hydrophobic boundary region of the cell membrane, must have happened, addressed phenomenologically in liposome binding assays.

## 2. Materials and Methods

### 2.1. Detection of In Vitro Liposome Bound Colchicine

As it is part of a patented technique, direct detection method (DDM) [10], we shall avoid presenting the details here, but can be found in our earlier publication [44]. The DDM consists of a novel experimental method which helps detect MAAs directly at the membrane. The methods applied for constructing liposomes, incubating them with drugs, and separating the unbound drugs from liposome bound ones have been explained in our previous publications [45,46]. Chloroform from lipid/cholesterol stock in chloroform was evaporated using a stream of nitrogen or argon. The dried chloroform-free lipids (dioleoylphosphatidylcholine (DOPC)) or lipid/cholesterol mixture was dissolved in experimental buffer (buffer: 0.5 M NaCl, 10 mM HEPES, pH 7.4), and 0.5 mL of each solution was transferred to an Eppendorf tube. Col (from Sigma, St. Louis, MO, USA) stocks were prepared in dimethylsulfoxide (DMSO) (from Burdick and Jackson, Muskegon, MI, USA) (4 mg/mL or 7.1 mM), diluted in buffer for further use (1 mg/mL). Appropriate amount of Col stock was added to the Eppendorf tube, considering concentrations 0, 1, or 100 μM Col to be achieved in the final liposome incubating buffer inside the Eppendorf tube. We then centrifuged the Eppendorf tubes to pellet lipid and the bound drugs using Hettich Rotina 35R (Hettich America LLP, Buford, GA, USA) at 19,520× *g* for 1 min. Supernatants (buffer and the unbound Col) were then removed. We again added 0.5 mL of experimental buffer into each Eppendorf tube, resuspended the lipid, and centrifuged them again to pellet lipid and the bound drugs and the supernatants were again removed. This process was repeated three times to effectively remove the unbound drugs. We then added 0.5 mL of methanol into the glass tube (to be used for Col detection) containing bound Col⁄lipids/liposomes, being dissolved using appropriate stirring for experimental detection of Col. Let us call this solution ‘B’. Following strategies adopted in DDM [10,44], the detection of bound drugs in the liposome was performed. The volume of solution B is always matched with that (0.5 mL) of liposome incubating solution to mutually normalize Col concentrations in both solutions before performing their detection. The absorbance spectroscopy was then applied to detect the lipid-bound Col (from Sigma-Aldrich) in mole (M) fraction; CDs in vitro DOPC or DOPC:cholesterol (2:1 molar ratio) liposome systems [19]. In this method (especially in the liposome experiment technique), the liposome-bound colchicines were detected (after washing the liposome with aqueous buffer at least three times from the rest of the solution containing unbound colchicines in the aqueous phase (solution UB) (see [10]). A NanoDrop (from ThermoFisher Scientific) or a Nanophotometer (from Implen GmBH) was used to get the absorbance spectra specific for the drug. The wavelength (λ_MAA_) of the spectrum is MAA specific. For example, λ_colchicine_ = 243 nm for colchicine (see Sigma-Aldrich manual). The spectroscopy was performed and the quantification of the colchicine concentrations was made in both solution B and UB [10]. From their detected concentrations the molarities of both lipid bound and lipid unbound colchicines in the incubated tubes were calculated.

### 2.2. Cell Culture and Treatment of Cells with Colchicine

Human colorectal adenocarcinoma cells, LoVo, were maintained in Dulbecco’s modified Eagle’s medium (DMEM; Gibco), and supplemented with 10% fetal bovine serum (FBS; Gibco) and 50 units/mL penicillin-50 μg/mL streptomycin (Gibco). At 75–80% confluence, cells were treated without (control) or with Col at various concentrations. Here two physiologically relevant Col concentrations, 1 μM and 100 μM, were considered and the Col-treated cells were incubated for 24 h. Cells were then trypsinized and washed in phosphate-buffered saline (PBS; Gibco), and counted using a handheld automated cell counter, Scepter (Millipore) fitted with 60 μm sensor to a final concentration of 5 × 10^5^ cells/mL in PBS. Cells were then ready for AFM investigations.

It would be interesting to compare with a noncancerous cell, however, each cell type in the human body has distinct lipid profiles of the plasma membrane depending on the tissue/organs they make and the different functions they carry out. Cancer cell lipid composition not only differs from noncancerous cells but also varies between different malignancy types. There is no specific lipid profile that can differentiate between cancerous cells from noncancerous cells since malignant cells are very dynamic and its lipid composition fluctuates with its progression to invasion and metastasis. Thus, performing similar experiments with either normal cells or different malignant cells having different lipid compositions might produce varying outcomes. Moreover, the colorectal adenocarcinoma cells used in our study is of epithelial origin and obtaining normal colorectal epithelial cells for comparison is extremely difficult. Therefore, in our biophysics focused model demonstration, we judiciously picked only one type of cancerous cells here. However, in the future we may plan to investigate and compare among various cell types.

### 2.3. AFM Experimental Methods

In AFM, a sharp tip was mounted to the end of a cantilever which was flexible. The tip was brought in contact with the sample surface. The surface of the sample was then scanned with the tip and thus the images were achieved. During scanning the tip over the surface, the force between the tip and the surface leads to the bending of the cantilever. Consequently, deflection is detected by a laser beam which is reflected at the end of the cantilever onto a sensitive detector. Three-dimensional (3D) topographic images of the sample surface were obtained by plotting the deflections as a function of positions along with the X-Y coordinates. A feedback loop was used to maintain a constant mode of deflection and the topographic information was obtained from the vertical displacement of the cantilever (for details, see [13,14,15]).

To study the surface morphology of cells, a few drops of cell solution were firstly cast onto chemically and ultrasonically cleaned SiO_2_ substrates and spin-coated (3000 rpm for 30 s). To remove the thermally unstable molecular conformations, the samples were heated at 35 °C for 30 min. After that, the samples were loaded into the microscope. Before imaging the surface by AFM, an optical image of the tip position and cell distribution on the surface was recorded. Two different AFMs were used: Tapping Mode of Bruker MultiMode 5 AFM (TM-AFM) for investigating the cell surface morphology and QNM Mode of Bruker MutiMode 8 AFM (QNM-AFM) for measuring the adhesion forces (the details on both AFM techniques are provided in the Supplementary Materials).

### 2.4. NCs Considering Relevant Physiological Parameters

NCs were performed based on a theoretical platform created by us recently [8,24]. A candidate drug in a drug or lipid cluster directly interacts with a nearest-neighbor drug or lipid by Coulomb forces and this nearest-neighbor drug/lipid interacts directly with the next-nearest-neighbor drug/lipid, but this second drug’s/lipid’s interaction with the candidate drug/lipid is screened by the candidate drug’s nearest-neighbor drug/lipid. Similarly, the subsequent third-, fourth-nearest neighbor, etc. drug/lipid interacts with the mentioned candidate drug but that their interactions experience second, third, etc. order screening, respectively. This SCI takes the following form, as explained in details in [8,24]

(1) $$V_{sc}(\vec{r})=\int d^{3}kExp\{i\vec{k}.\vec{r}\}V_{sc}(\vec{k})$$

which is in Fourier space [8,24]

(2) $$V_{sc}(\vec{k})=\frac{V(\vec{k})}{1+\frac{V(\vec{k})}{2\pi k_{B}T}n}$$

where *V*(*k*) = (*1*/*ε*_0_ *ε_r_)q_D_q_L_*/*k*^2^ is the direct Coulomb interaction between drug (D) (charge *q_D_*) in a drug/lipid cluster and the nearest-neighbor drug/lipid (lipid charge *q_L_*), *k* is wave number, *T* is the absolute temperature of the aqueous phase, *n* is the density of particles, *k*_B_ is Boltzmann’s constant. *ε_0_* is the dielectric constant in vacuum, *ε_r_* is the relative dielectric constant where interaction takes place.

The Fourier transformation of the Equation (1) was performed using Mathematica 9.1 program-based NCs (for detailed methodology, see [8,24]) to detect values of drug binding energy δG between drug–drug/lipid in drug or lipid clusters. δG is first, second, etc. order terms in the expansion of *V*_sc_(*r*) for the hydrophobic mismatch to be filled by single, double, etc. particles representing first, second, etc. order screening, respectively, in the cluster. In our current investigation *q_D_* and *q_L_* are charges of Col (*q*_Col_) and PC (*q*_PC_), respectively, in the aqueous phase. *K* ≈ 2π/a, a is the average Col-Col or Col-PC distance in Col or PC clusters while both appear as nearest neighbors to each other. The values could be assumed as average Col dimension (~1 nm) or PC headgroup dimension (~0.77 nm), respectively. In reality, we might also need to take into consideration many other parameters related to, e.g., variations in the membrane’s electrical conditions, the presence of hydrocarbons within, etc. *n*, the particle (drug or lipid) density, was chosen ~1/60 Å^2^ (for a~0.77 nm). *k*_B_*T* ≈ 1.38 × 10^−23^ Joule/*K* (300 *K*), *ε_0_* is the dielectric constant in vacuum, *ε_r_* (~2) is the relative dielectric constant inside the membrane [41].

## 3. Results

**Figure 1:**
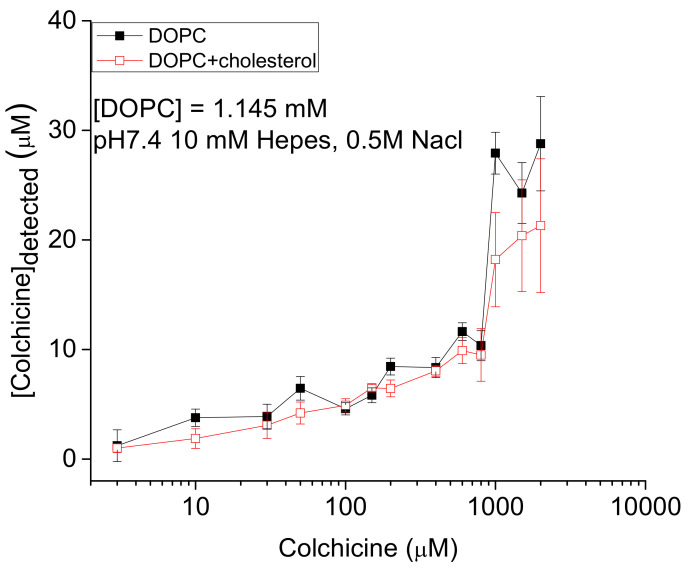
Liposome adsorbed colchicine concentration versus liposome incubating colchicine concentration plots. y-axis values are the (liposome adsorbed) concentrations of colchicines (in solution ‘B’, see Section 2.1) detected directly in the liposomes using DDM method, after the liposomes being incubated with various colchicine concentrations (x-axis values) in aqueous phase. Two sets of data (mean ± S.D., n = 5) represent two types of DOPC liposomes, without and with cholesterol (DOPC:cholesterol = 2:1, molar ratio), respectively.

Colchicine, bound to the in vitro constructed liposome system, has been quantified in Figure 1. There is a systematic trend of increased liposome binding as the colchicine concentration in liposome incubating buffer increases. These results demonstrate that a direct mechanism of liposome binding of drugs is active because the liposome detected colchicines are permanently attached/adsorbed there. Otherwise, the drugs should have been released to the solution while the liposomes being washed a couple of times using buffer [10]. The presence of the cholesterol in liposomes does not qualitatively modify the liposome adsorption process though quantitatively perturbs the adsorption of drugs only modestly. The cholesterol effects may be compared with other previous biophysical studies on the effects of cholesterol suggesting for drugs’ (e.g., antimicrobial peptides) effects to be less in cholesterol-containing lipid membranes than observed in cholesterol-free bilayers [23,47,48].

In cell studies, we first performed a control AFM experiment on a single bare cancer cell. The characterization of the cell is explained in Supplementary Figure S2 (see ‘Independent Cell and Colchicine Characterization’ in Supplementary Materials). AFM topography images (height, amplitude error, and phase) of a cancer cell on SiO_2_ substrate are presented in Supplementary Figure S3A (top panel). A few visible small blobs in the background of the figures could be reminiscent of solvent molecules used to prepare the solution of the cells.

Our objective was to inspect whether the colchicine molecules were physically bound to the cancer cell membranes. The colchicine-treated cells were deposited onto the SiO_2_ surface for recording AFM images. AFM topography images of a single colchicine-treated cell are presented in the bottom panel of Supplementary Figure S3A. Here we see that the shape of the cell is distorted. This could be due to the surface induction of colchicine into the cell. It is more visible from the bottom panel phase image (see Supplementary Figure S3A(c)) that the cell is sitting on the incubating liquid. In contrast to the top panel, the bottom panel image (Supplementary Figure S3) shows a few blobs of different sizes on the cell membrane. This is visible in 3D images of the cell (see Supplementary Figure S3B).

**Figure 2:**
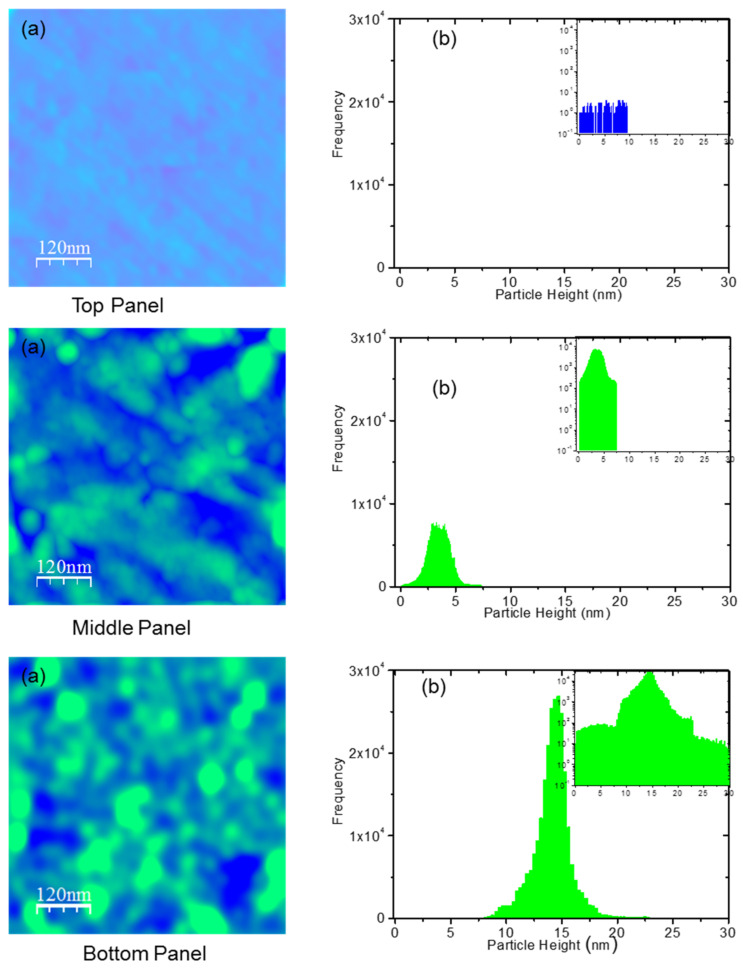
Top-left panel (**a**), high resolution TM-AFM image of a control (colchicine untreated) cell surface. Top-right panel (**b**), histograms presented here are based on counted clusters on the section of cell surface shown in the left panel (**a**). Each histogram represents the number of counted clusters (called frequency, let us assume it as *f*_i_, plotted along y-axis. i = 1, 2, 3, …, etc.) versus a height profile (known as particle height, Figure 2b (top-right panel), let us assume it as *h*_i_, plotted in nm scale along x-axis) shown in Figure 2b. As mentioned in Supplementary Figure S5, the height profiles are recorded at the centers of the corresponding clusters (see the left panel), Figure 2a. Middle and bottom panels present data for cells treated with 1 and 100 µM colchicine, respectively. The higher presence of clusters (in green color) on blue background is visible. We repeated the experiments three times but inspected no substantial changes in the distribution patterns presented in the right panels. Origin 9.1 was used to plot histograms after detecting the numbers using program WSxM. The origin of the detected few events (visible in log plots, see in insets of Figure 2b) for untreated/control cell surface is nothing but noise.

The heights of different blobs on the colchicine treated cells were measured using high resolution AFM height images (see Figure 2, left panels). For observing a few colchicine clusters and the related height profiles relative to the single colchicine size, see Supplementary Figures S4 and S5. We observe the particle and cluster distribution here by plotting the frequency of clusters in a histogram plot, see Figure 2 (right panel). We calculate the total number of the cell surface clusters of the colchicine molecules (*N*_cluster_) using following formula

(3) $$N_{\mathrm{cluster}}=\overset{n}{\underset{i=1}{\sum }}f_{i}$$

The total number of cell surface adsorbed colchicine molecules (*N*_col_) can be deducted using the following formula

(4) $$N_{\mathrm{Col}}=\overset{n}{\underset{i=1}{\sum }}f_{i}h_{i}/a_{\mathrm{Col}}$$

By assuming colchicine molecules as hard spheres we can calculate the total colchicine molecules accumulated along the line of a cluster height *h_i_* to be *h_i_*/*a*_Col_, where *i* = 1, 2, 3,…, etc.

**Figure 3:**
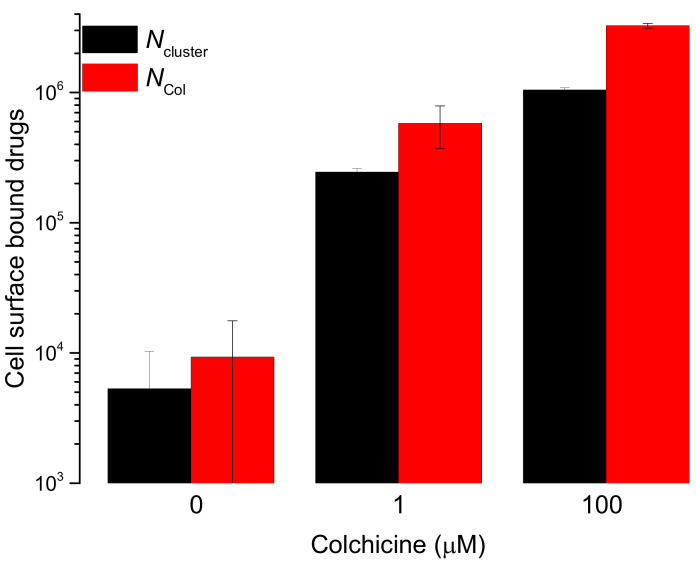
Cell surface bound/adsorbed colchicine molecules (*N*_Col_) and cell surface clusters of the colchicine molecules (*N*_cluster_) have been plotted (y-axis) as histograms at the cell treating 0 (control), 1 and 100 µM colchicine concentrations. Both sets of data represent the mean ± S.D., n = 3. The number each histogram represents is the counted number of clusters against a particle height profile (see Figure 2b and Supplementary Figure S5b). The height profiles are recorded at the centers of the corresponding clusters in Supplementary Figure S5a.

*N*_cluster_ and *N*_Col_ have been plotted in Figure 3. The trend of cell surface adsorption of colchicine shows strong tendency of saturation between colchicine concentrations 1 and 100 µM.

The size and number of colchicine clusters increase with increasing colchicine concentrations (Figure 2a). The particle heights *h_i_*s (x-axis values on which histograms stand, Figure 2b) also increase quickly with increasing concentration of colchicine, reflected in the histogram positions in the wide range in x-axis values. The detected particle height values in clusters are measured relative to the dimension of the single colchicine molecule which is of the order of 1.1 nm (Supplementary Figure S4). The colchicines are thus mostly detected in clusters having various dimensions on the surface of the cells being treated with high colchicine concentrations.

**Figure 4:**
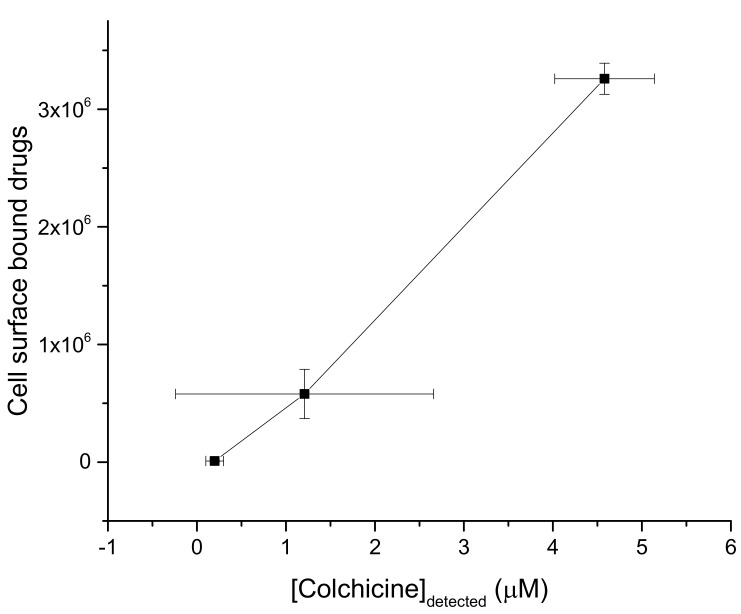
Comparison between number of cell surface bound colchicine molecules versus liposome adsorbed colchicine for three common aqueous Col concentrations in both investigations, namely 0 (control), 1 and 100 µM (from the low x-y coordinates to the higher ones in the graph). The data represent for the experimental repeats, n = 3 and 5 for cell surface binding (mean ± S.D., n = 3) and liposome binding (mean ± S.D., n = 5) data, respectively. In this figure, y-axis values represent the y-axis values for *N_Col_* taken from Figure 3 and x-axis values represent the y-axis values for colchicine concentrations in DPPC liposomes taken from Figure 1, respectively.

We wish to compare the cell surface-bound colchicine molecules and liposome adsorbed colchicine. These two sets of data may not be directly correlated as the data are produced in apparently two different membrane environments, namely the actual cell surface having a lot of other materials besides those considered in controlled in vitro environment constructed liposomes. However, the comparison may still hint at some actual scientific understanding regarding lipid interaction-based drug adsorption. The plot in Figure 4 suggests that the data produced in these two physiological environments are almost linearly correlated. This hints perhaps for the common type direct ‘lipid interaction’ or ‘lipid association’ driven adsorption and/or accumulation of drug molecules in membrane regions.

### 3.1. Strong Adhesion between Colchicine Cluster and Cell Membrane

**Figure 5:**
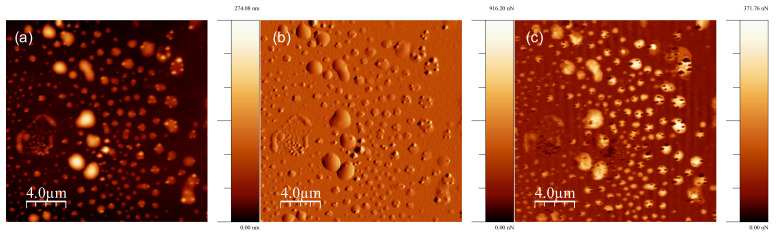
QNM-AFM image showing the distribution of cells with colchicine binding at a large scale on SiO_2_ substrate: (**a**) AFM height image, the height of the cell is represented in color scale 0 (dark fields)–274.08 nm (light fields). (**b**) AFM force error image, the force of the cell is represented in color scale 0 (dark fields)–916.20 nN (light fields). (**c**) AFM adhesion image of the cell, the adhesion of the cell is represented in color scale 0 (dark fields)–371.76 nN (light fields).

**Figure 6:**
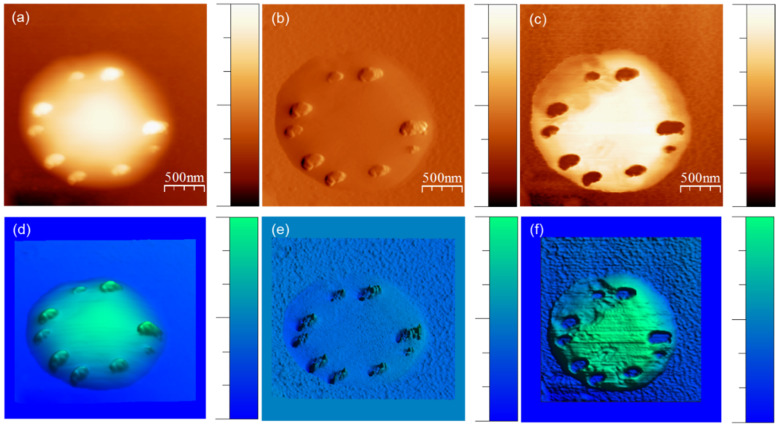
QNM-AFM images showing colchicine bound to cell on SiO_2_ substrate. Top panel, (**a**) height image, the height of the cell is represented in color scale 0 (dark fields)–175 nm (light fields). (**b**) Force error image, the force of the cell is represented in color scale 0 (dark fields)–326 nN (light fields). (**c**) Adhesion image of the cell, the adhesion of the cell is represented in color scale 0 (dark fields) –206 nN (light fields). The corresponding 3D images are presented in bottom panel, (**d**–**f**), respectively.

Adhesion force was measured at every pixel of the topography map by using QNM-AFM. This measurement helps understand the interaction between colchicine molecules and cell membranes. The samples were completely dried, consequently, no liquid was present on SiO_2_ surface and colchicine molecules were observed in clusters on the cell surface (see Figure 5). For measurement/imaging techniques we had to compromise with the aqueous cell phase as we dried the sample, but the overall physics of strong binding (see numerical computation results, presented later) is thought to be intact. High resolution QNM-AFM images of a single cell treated with colchicine are presented in Figure 6.

Quantitative adhesion force (F_ads)_ between probe and surface (SiO_2_, drug, and cell membrane) was extracted from Figure 6c by using a NanoScope analysis software 1.5. This adhesion force was then converted into adhesion energy ($\Gamma_{\mathrm{ads}})$ using equation, $\Gamma_{\mathrm{ads}}=\frac{F_{\mathrm{ads}}}{2\pi R}$ [42] where *R* is the radius of the AFM tip, approximately 8 nm. The adhesion energy between drug and cell membrane ($\Gamma_{\mathrm{drug}–\mathrm{cell}})$ has been calculated by using the Equation [43]

**Figure 7:**
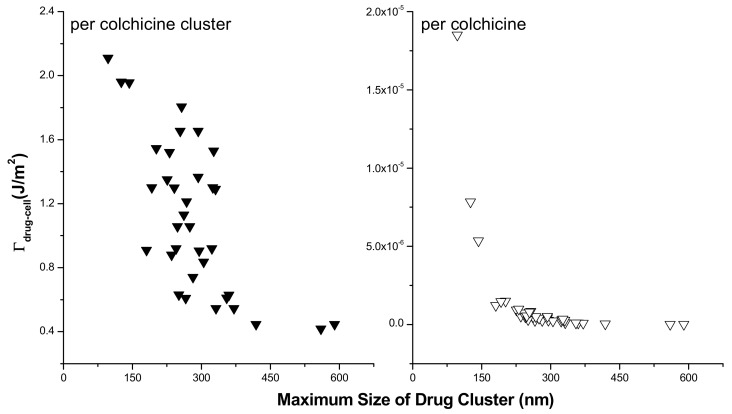
Adhesion energy between drug and cell surface is plotted against drug cluster size. Left panel: energy per drug (colchicine) cluster. Right panel: energy per drug molecule in a cluster.

(5) Γdrug–cell=2γdrug×γcell12

here γ is the surface energy. Using the same equation, we calculated the surface energy of AFM tip, $\gamma_{\mathrm{tip}}=0.252\;J.m^{-2}$ where surface energy of SiO_2_ surface was used, $\gamma_{\mathrm{SiO}2}=0.15\;J.m^{-2}$ [43]. The adhesive energy $\Gamma_{\mathrm{drug}–\mathrm{cell}}$ between colchicine clusters and cell membranes has been calculated from quantitative adhesive force measurements by AFM on 35 clusters over 97–590 nm size distribution, chosen randomly from five different cells. $\Gamma_{\mathrm{drug}–\mathrm{cell}}$ is plotted against cluster size in Figure 7. $\Gamma_{\mathrm{drug}–\mathrm{cell}}$ is observed to decrease with increasing cluster size. We then calculated $\Gamma_{\mathrm{drug}–\mathrm{cell}}$ per single colchicine drug and found that the adhesion energy per colchicine decreased with increasing cluster size before gradually saturating beyond 225 nm cluster size (see Figure 7).

### 3.2. Modeling the Distribution and Computing the Energetics of Drugs on the Cell Surface

**Figure 8:**
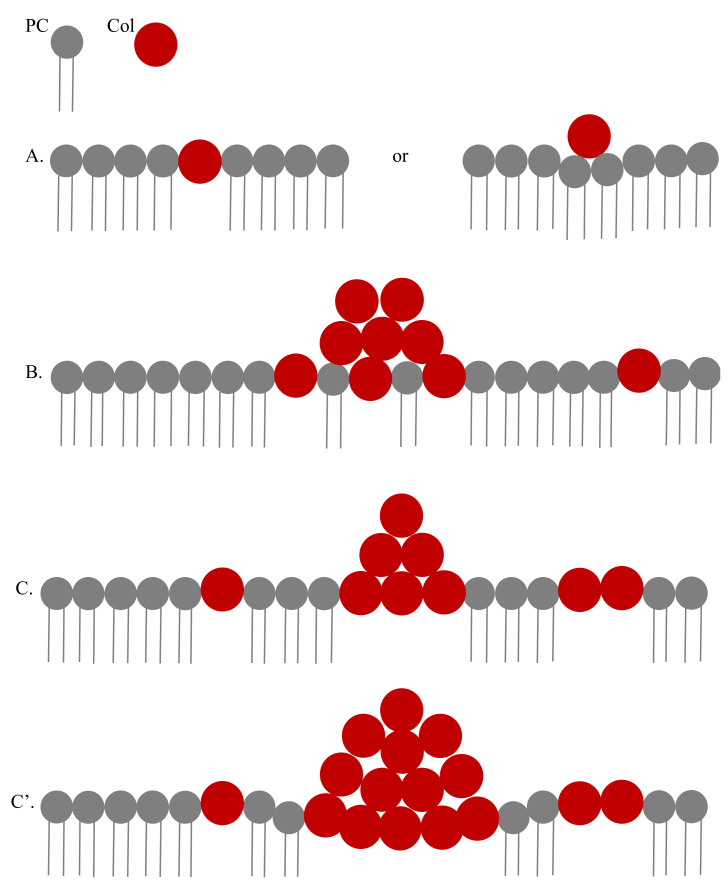
The distribution of drugs on the cell surface has been modeled. (**A**) Top panel demonstrates the single drug adsorption on the cell surface what we brand as the drug in the lipid cluster (right side confirmation is also supported due to lipid membrane elasticity [4] which ensures modest lipid monolayer bending before actually breaking or rearranging the structure as shown in left side). (**B**,**C**,**C’**) demonstrate the creation of drug clusters on the cell surface. (**B**) shows that the drugs and lipids may coexist inside cluster in its 2D base (cross-sectional view over one lipid layer is shown here) aligned with the surface of the membrane monolayer, whereas (**C**). suggests that the drug cluster expels lipids totally, the scenario we have branded as drugs in drug clusters. Drug clusters in both distinguishable conditions presented in (**B**,**C**,**C’**) may push the monolayer a bit towards the hydrophobic membrane core, depending on the amount of downward acting gravity which is predicted (not calculated here) to be higher for larger clusters (see (**C’**)). (**C**,**C’**) represent identical conditions, except the latter representing a bit bigger than the former cluster. The different types of drug clustering may also concomitantly exist on different locations of the cell surface. As both drugs and lipids are continuously dynamic with the time scale of molecular movement of the order of millisecond (ms) or less (maybe considered equivalent to the ion pore stability as detected in various studies including e.g., in [8,24]), the whole process follows principles of Statistical Mechanics. For simplicity, we have just sketched a lipid monolayer although cell membrane is a lipid bilayer. However, it is also true that while penetrating into the cell through lipid membrane from cell surface drugs face cell surface lipid monolayer, as we have modeled here. The CD-induced pore formation certainly involves both monolayers (models are presented in [1,2]).

Regarding AFM detected drug clusters on cell surface it is to mention that Col cluster means a domain of Col molecules in which a Col is surrounded by neighboring Col molecules. Col treated liposome experiments suggest substantial Col molecules adsorption by lipid membranes. This drug adsorption is reflected in AFM data suggesting cell surface binding of Col molecules in independent and cluster formats. The height profile plot in Figure 2 suggests that the Col cluster may appear with a height profile too, meaning the cell surface distribution of Col molecules in a Col cluster occurs three-dimensionally (3D). This Col cluster may take such a shape (internally packed with Col molecules) on the cell surface that it keeps vanishing along x-y dimensions, as its z-dimension increases towards the peak value of the height of the cluster. Whereas, regarding a Col in a PC cluster, we mean nothing but the presence of a single Col on PC membrane-more like free drug movement on the cell membrane. An intermediate state may also be considered where domains consisting of mixed Col and PC molecules are formed. However, in this mixed domain, PCs (due to their undeniable hydrophobic pull) exist only on the two-dimensional (2D) x-y plane of the cell surface, but Col molecules may remain anywhere including along the z-axis inside the domain. We have modeled the scenario in Figure 8.

**Figure 9:**
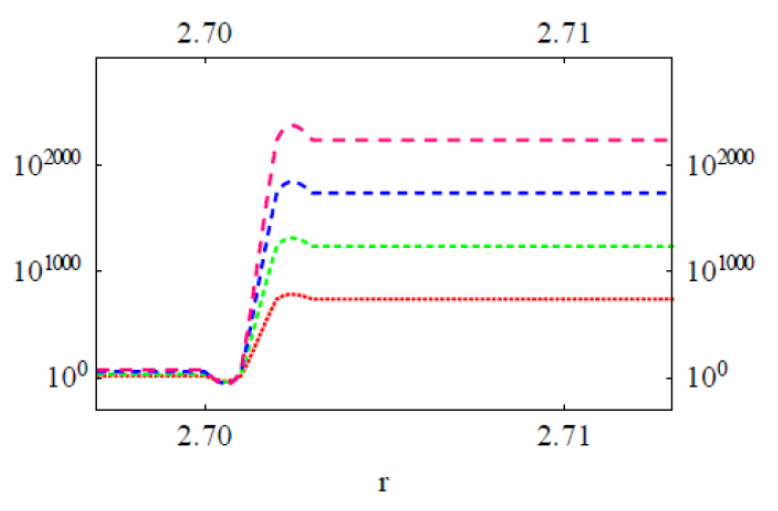
Numerically computed scale free energy versus reaction coordinate r plots of Equation (1). Col in Col cluster (top panel) and Col in lipid (e.g., PC) cluster (bottom panel). In both panel figures there are 4 curves representing energies of four SCI orders first, second, third, fourth (bottom to top). Here a = 1 nm, *q*_Col_*q*_PC_ = 10^−2^*e*^2^, as PC is nearly charge neutral, but MAAs are negatively charged in physiological aqueous phase (see [44]). *e* is the electron charge.

**Figure 10:**
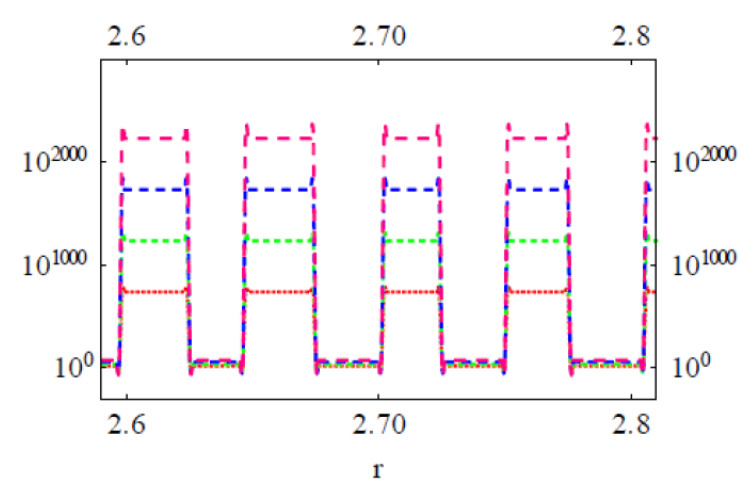
Numerically computed energy versus reaction coordinate plots of Equation (1). Col in Col cluster (top panel) and Col in lipid cluster (bottom panel). In both panel figures there are four curves representing energies of four SCI orders first, second, third, fourth (bottom to top). Here a = 1 nm, *q*_Col_*q*_PC_ = 10^−2^*e*^2^. The back-and-forth transitions for any SCI order shown here happen between two energy states; the pattern of the transition is shown (from lower to higher energy state transition) in expanded scale plots in Figure 9.

We have performed a series of NCs considering the drug distributions in Col and PC domains on the cell surface (see Figure 8). The approximate consideration of 2D treatment for a Col cluster is possible when the cluster size is small (the height of the cluster approaches the lowest possible value). Numerically computed energy versus reaction coordinate plots of Equation (1) have been presented in Figure 9 and Figure 10. All curves have two energy states, namely low energy state (G_I_) and high energy state (G_II_) (see Figure 9 where we have presented single transitions for all 4 SCI orders). The curves undergo transitions (accounting for drug–drug/lipid association ↔ dissociation) between these two energy states at various values of r (see Figure 10). δG =│G_I_˗G_II_│. As values of G_I_s for all SCI orders are almost the same, the difference in δG arises from increasing (on a geometric order) values of G_II_s with increasing SCI order.

**Figure 11:**
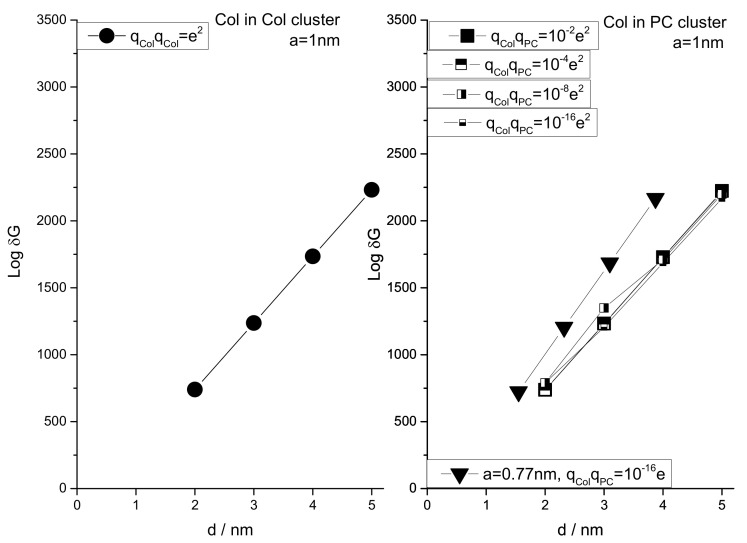
Energy for inter particle distance for four SCI orders. The inter particle separation *d* is deducted for each SCI order considering a close drug–drug (left panel) or drug–lipid (right panel) packing conformation.

**Figure 12:**
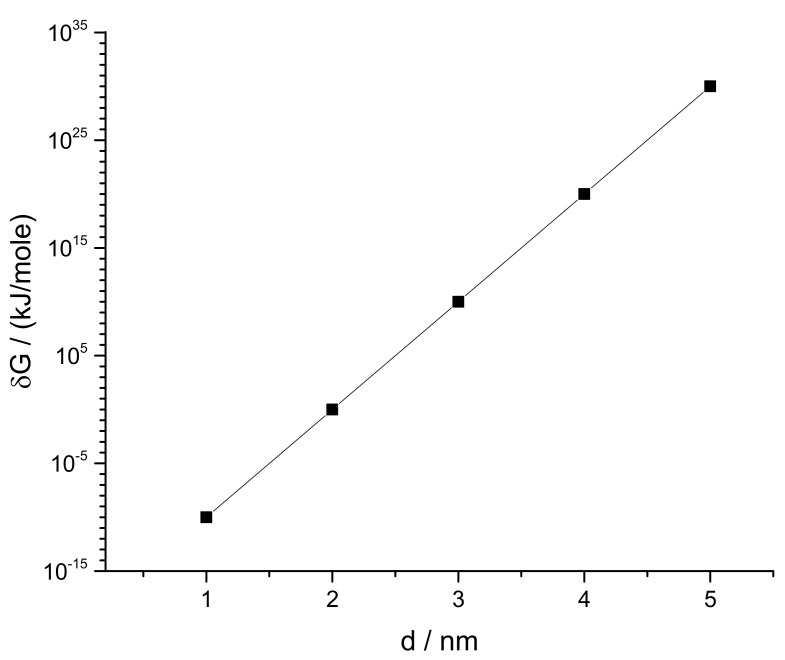
Energy in kJ/mole for inter particle distance, following ad hoc assumptions (see Materials and Methods: NCs considering relevant physiological parameters) [8]. Values of δG is found in order detected in MD simulations for a drug–lipid pair interaction where d ≤ 2 nm [2].

δGs for all four SCI orders have been deducted and their logarithmic values are plotted against corresponding inter particle separations in Figure 11 for different particle charge (relative charges of Col and PC) and lattice constant a (Col and PC dimensions) conditions. δG is observed to increase exponentially with increasing SCI order (or corresponding increasing inter-particle separation *d*) for drug interactions in both drug and lipid clusters. Values of δG for drug clusters are higher than that for lipid clusters. Another important observation is made that as the value of lattice constant (a) decreases (e.g., from 1 nm to 0.77 nm) the value of δG increases for corresponding values of *d*, due to perhaps closer packing of particles (checked for q_Col_q_PC_ = 10^−16^*e*). The formation of bigger drug clusters accounting for the requirement of higher SCI orders in drug–drug interactions is harder [8]. Concomitantly, the formation of smaller drug clusters is easier and is expected to get formed with a higher probability than that of bigger drug clusters. Similarly, binding of a drug with distant lipids requiring higher SCI orders in drug–lipid interactions is less likely, so the probability of existing single drug with the minimum (low order SCI) cell–surface interactions is higher. Figure 12 presents the values of δG in kJ/mole for Col–PC interactions (for a = 1 nm and q_Col_q_PC_ = 10^−2^*e*). It seems that the required free energy change for inter-particle association↔dissociation goes fast beyond the physiologically relevant energies (~kJ/mole in single-digit [22]) as *d* increases. δG is found to be in order that was detected in MD simulations for a drug–lipid pair interaction where *d* ≤ 2 nm [2]. The binding energetics of drugs and lipids on cell surface demonstrates that the probability of observing single colchicine on the cell surface is highest, and the probability of finding drug clusters decreases as cluster size increases. This molecular-level energetics supports the AFM data (see Figure 2).

**Figure 13:**
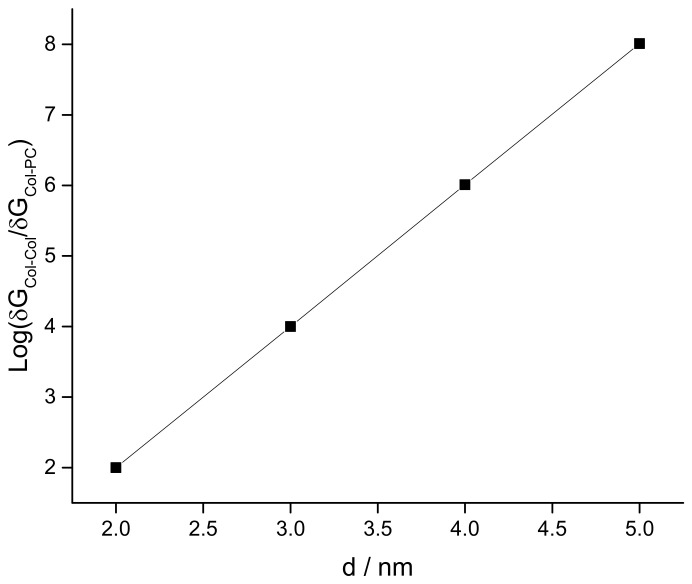
Plot of Log(δG_Col-Col_/δG_Col-PC_) addresses on relative energetic cost a drug experiences to fall into a drug cluster from lipid bound state. δG_Col-Col_ and δG_Col-PC_ are the values of δG for Col in Col cluster and Col in PC cluster, respectively (plotted independently in Figure 11). A = 1 nm.

The plot of Log(δG_Col-Col_/δG_Col-PC_) addresses the relative energetics any drug experiences in a drug cluster in comparison to that in a lipid cluster (see Figure 13). Values of Log(δG_Col-Col_/ δG_Col-PC_) increases proportionally with *d*, suggesting heavier energetic costs for the transition into drug cluster while drugs interact over higher-order screening. We may hypothesize that the single drug on the lipid membrane causes the initiation of drug cluster which is the smallest theoretical size limit of a cluster. Adding more and more drugs into the cluster requires exponentially increasing higher-order energetics. The first collapse of the adhesion energy (accounting for stronger binding) between drug and cell surface with increasing cluster size (Figure 7 (right panel)) hints for the requirement of higher-order change of energetics for drugs’ association↔dissociation, an experimental finding gets explained in the trend found in numerical investigations on SCI model calculations (Figure 13).

## 4. Discussion

AFM data and phase diagrams demonstrate the cell surface binding of CDs. Quantitative cell membrane adsorption of drugs is demonstrated in liposome experiments, which directly gets imaged on cell (membrane) surface in AFM imaging techniques. Increasing drug concentration in buffer causes increasing liposome binding and cell surface adsorption. The ratio of two quantitative membranes (liposome and cell surface) associations has been found to follow a linear progression (see Figure 4). These data hint at a possible general direct lipid interaction or association-driven adsorption and/or accumulation of drug molecules in membrane regions. The cell surface drug adsorption follows strong statistical nature. Drugs appear independently (associated with membrane lipids) as well as in clusters (associated among drugs) with various cluster sizes in their cell-surface bound states. The frequency and size of clusters increase with the cell incubating drug molarity, a trend also found for various peptides and chemotherapy drugs while inducing ion pores/channels (regarding, e.g., pore density and/or membrane conductance) into planar lipid bilayer membranes [1,2,8,22,23]. Phenomenological lipid membrane adsorption (addressed in liposome experiments) and its regulation due to the effects of cholesterol are both demonstrations of cell surface (membrane) accumulation of drugs in the alternative, yet cell membrane mimicking in vitro constructed controlled membrane systems. The measurement of the cell surface cluster heights helps predict the number of colchicine molecules accumulated there. The creation of drug complexes on the cell surface is understood to be due to physical binding. The average adhesive energy depends on the drug cluster size. These data altogether hint at the creation of special physical structures following specific laws of physics as a result of the combined clustering of drugs with cell surface constituents, namely cell membrane lipids and proteins. CDs are discovered to interact with lipids due to their charge properties [2]. In MD simulation of drug–lipid complexes, van der Waals (vdW) and electrostatic (ES) interactions are primarily detected, providing vdW and ES energies, respectively (see Supplementary Figure S6) [2]. Theoretical screened Coulomb and vdW interaction formalisms have been developed to address the mechanisms of ion pore formation due to MAAs—such as various peptides, CDs, etc.—inside lipid bilayer membranes [8,24,44]. These charge-based interactions and the related energies depend largely on the participating agents’ mutual organizations, yet following universal molecular mechanisms (see [44]). The theory, NC, and MD simulation-based information together suggest that a primarily charge property-based SCI interaction portfolio may be active behind the creation of a cluster among the participating agents and that the cluster formation perhaps eventually leads to the creation of the MAA induced ion pores [8]. We have demonstrated here the general drug adsorption of liposomes and the formation of drug molecule clusters on a cell surface. General cell surface binding and cluster formation phenomena may not directly prove that these molecular processes are the sole causes of ion pore formation inside the cell membrane. However, the nature of such drug adsorption mechanisms of the liposome membrane and on the cell surface may suggest that following the slow chemical process of drug adsorption the system experiences fast perturbations in the physical properties (localized mechanical and electrical) of the bilayer, thus may lead to causing the energetics and corresponding conformations to be regulated [8]. This is evident in the pattern of energies and stability of drug–lipid complexes shown in Supplementary Figure S6 [2]. The reasonable minimum drug–lipid binding energy conformation may statistically appear due to phenomenological drug binding and clustering on cell surface regions. A clear hint of such kind is automatically revealed in the trend of adhesive energy measured/calculated and free energy of binding numerically computed here. The statistical membrane adsorption and possible delivery of drugs, and related energetics may also be regulated due to the modulation of membrane chemical environment [49,50]. The general drug adsorption is tested in experiments on liposomes without and with the effects of cholesterol. Modest modulation in drug adsorption has been found due to cholesterol (which naturally promotes bilayer plasticity) unlike the enhancement in drug adsorption observed recently due to bilayer physical property modulating (bilayer elasticity promoting) amphiphiles capsaicin and triton X-100 [51] and small cyclic decapeptide gramicidin S [52] in vitro constructed lipid bilayer or liposome systems.

The membrane interaction/adsorption of these pore-forming CDs may also regulate membrane physical properties—such as membrane fluidity, stiffness, thickness, even membrane phase properties, etc.—and vice versa like hinted for other membrane-active peptides [53,54,55,56]. To conclude we certainly need further studies. Although modest changes in these membrane physical and phase properties may happen, e.g., colchicine treatment has been found to yield entropy increase and produce higher disorder of the macrophage membrane [57], such changes may not contribute primarily in causing the drastic phenomenological changes in cell surface morphology we observed due to CDs using AFM imaging: up to 23 nm cluster height profiles observed with drugs (Figure 2b-bottom panel) compared to around 7 nm without (Figure 2b-top panel). Therefore, cell surface drug accumulation is the only prediction we can make with confidence. The adhesive energy behind drug accumulation and clustering on the cell surface is measured to be significant. Its drastic decreasing trend with increasing cluster size hints at the requirements of higher-order energetic penalty for ensuring binding condition in bigger clusters satisfying a fundamental physics concept which equivalently suggests that bigger clusters should represent for deeper potential wells [58]. Thus, a drug molecule—to get trapped inside the deeper well—must undergo huge energetic changes which recall various SCI orders in the interaction portfolio developed using SCI modeling [8,24]. Our current study conclusively provides evidence for the phenomenological membrane adsorption of colchicines, statistical cell surface distribution and energetic binding of the drugs causing concentration-dependent drug clustering with various sizes (heights and diameters), leading to possible pore formation across cell membranes.

## 5. Conclusions

CD molecules statistically bind on cell surfaces randomly as individual particles or in clusters. The liposome and cell surface drug adsorption and the detected energetics of permanent type drug binding may be suggestive of strong interactions of drugs with specific cell surface agents, including lipids specifically. These mainly charge property-based interactions and associated energetics perhaps play primary roles behind CD pore formation. Strong adhesive energy of drug molecules in drug clusters, vdW, and Coulomb or screened Coulomb interactions (comparable to other ion channels [2,44]) in drug–lipid complexes appear as premier mechanisms behind the cell surface binding of CDs. The lipid interactions of drugs are predicted as the major contributors in the cell surface binding energetics, but the broader picture is a bit complicated as the free energy per drug is dependent on whether the drug interacts with lipids independently or in a drug cluster. In adhesive energy measurements, we find a hint on it (Figure 7) while NC results explain this molecularly (Figure 13). Favorable energetics in adsorbed drug clusters may lead to the creation of ion channels [9,10]. This article demonstrates phenomenological liposome adsorption, cell surface binding, and clustering of chemotherapy drugs, and reveals novel picture on energetics behind lipid binding of drugs leading to pore formation in lipid membranes. This article will especially help the drug discovery scientists [10,44,59] to understand cell surface action and cytotoxicity of various agents.

## Data Availability

All relevant data have been incorporated in the article and/or in the associated Supplementary Materials (published).

## Supplementary Materials

The following are available online at https://www.mdpi.com/article/10.3390/membranes11070501/s1, Figure S1: CDs thiocolchicoside, a colchicine derivative (TCC) and taxol (TXL) (both at 90 μM) permeabilize lipid bilayer membranes by inducing nonzero current events, toroidal pores. Here, buffer pH = 5.7, applied trans bilayer potential V = 100 mV. Both traces were filtered at 20kHz, but the lower one shows higher noise due to its presentation (current axis) at amplified scale. pA for pico ampere current. For details see refs. [2,3]; Figure S2: Cell and colchicine TM-AFM image on SiO2 substrate: (a) Optical microscope image at large scale. (b) AFM height image of distribution of cells at a large scale. The height of the cells are presented in color scale 0 (dark fields)–293.04 nm (light fields). (c) AFM height image of distribution of colchicine (1 µM) molecules. The height of the colchicine molecules are presented in color scale 0 (dark fields)–7.33 nm (light fields). (d) A height profile recorded along the dashed line in Figure 1c; Figure S3A: TM-AFM image of a single cell on SiO2 substrate with and without colchicine binding: Top panel represents images of a bare cell (a) AFM height image. The cell height is presented in color scale 0 (dark fields)–390 nm (light fields). (b) AFM amplitude error image. The amplitude of a cell is represented in color scale 0 (dark fields)–157 mV (light fields). (c) AFM phase image of the cell. The phase of the cell is represented in color scale 0 (dark fields)–67 Deg (light fields); Bottom panel presents images of 1 µM colchicine treated cells. (a) AFM height image, the height of the cell is represented in color scale 0 (dark fields)–319 nm (light fields). (b) AFM amplitude error image, the amplitude of the cell is represented in color scale 0 (dark fields)–119 mV (light fields). (c) AFM phase image of the cell, the phase of the cell is represented in color scale 0 (dark fields)–65 Deg (light fields); Figure S3B: Three-dimensional (3D) TM-AFM height image of a single cell in Figure 1a (top and bottom panels) with and without colchicine binding on SiO2 substrate: (a) A single cell without colchicine binding. The height of the cell is presented in color scale 0 (dark fields)–390.72 nm (light fields). (b) A 1 µM colchicine treated cell. The height of the cell is presented in color scale 0 (dark fields)–327.70 nm (light fields). It is clearly seen that the cell is sitting on the liquid. Figure S4: The diameter (dcol) of a single colchicine molecule is approximately, dcol ≈ 1 nm. Minimum 1 nm and maximum 1.18 nm depending on the orientation have been measured using crystallography software ‘Diamond’. Left: A ball-stick representation of a colchicine molecule. Right: A space-filling representation of the same molecule. Hydrogen are omitted for clarity; Figure S5. (a) A high resolution AFM height image of a 1 µM colchicine treated cell wall bound to SiO2 substrate surface. The height of the cell wall is presented in color scale 0 (dark fields)–7.43 nm (light fields). (b) A number of height profiles recorded through the center of the circles marked in Supplementary Figure S5a. Here we marked the blobs (or clusters of colchicine molecules) of different sizes by circles of various colors. A number of height profile were recorded through the centers of the circles in Supplementary Figure S5a and presented in Supplementary Figure S5b, which ranges approximately between 1.1–5.5 nm. Therefore, it can be concluded that the colchicine molecules bind to the cell membrane either in single or cluster form; Figure S6: Here energies are detected from MD simulation on drug-lipid complexes. Simulations have been performed on two of the major cell membrane constituent lipids phosphatidylcholine (PC) and phosphatidylserine (PS) to address their interaction with cell membrane adsorbed CDs. In all four histogram plots (upper panel) of time versus *d*_drug-lipid_ thetime durations when drug/lipid stay together (height) within a distance (width) during 6 ns simulations are presented. Lower panels show the histograms of non-bonded van der Waal’s (vdW) energy (E_vdW_) and electrostatic (ES) interactions energy (E_ES_). To avoid color conflict E_vdW_ and E_ES_ are shown to occupy half-half widths though each half represents the whole width of the corresponding histogram. For details see ref. [3].
