# Supplementary material for: Cell Surface Binding and Lipid Interactions behind Chemotherapy-Drug-Induced Ion Pore Formation in Membranes
Source: Membranes (Basel). 2021 Jun 30;11(7):501. doi: 10.3390/membranes11070501 (PMC8304557; doi:10.3390/membranes11070501)
Supplement: Supplementary file 1 [file membranes-11-00501-s001.zip › membranes-1250346-supplementary.pdf]

membranes

Supplementary Material: Cell Surface Binding and Lipid Interactions behind Chemotherapy Drug Induced Ion Pore Formation in Membranes

Md. Ashrafuzzaman [1,*], Zahid Khan [2], Ashwaq Alqarni [1], Mohammad Alanazi [1,2] and Mohammad Shahabul Alam [3,4]

Supplementary AFM Techniques, Figures and Materials

Technique of TappingMode AFM (TM-AFM) for Cell Surface Morphology Study.

In order to study the surface morphology of cells, a few drops of cell solution was first casted onto chemically and ultrasonically cleaned SiO_2 substrates and spin coated (3000 rpm for 30 s). The sample was heated at 35 °C for 30 minutes to thermally stabilize the molecular conformations. The samples were then loaded into the microscope. Before AFM imaging, an optical image of the tip position and cell distribution was recorded. AFM measurements were performed by using a TM-AFM (Veeco V) with an antimony doped silicon cantilever (model: TESPA). Resolution for topography measurements were 512 × 512 points at 1 Hz frequency. AFM image figures were produced using the program WSxM [1]. The results were reproduced at least on 5 different cell samples for both control and colchicine treatments.

Technique of MutiMode 8 AFM (MM-AFM) for Adhesion Force Measurements.

Quantitative adhesion force images were recorded on a MM-AFM, Bruker. The Antimony (n) doped Si tip with spring constant of 200 N/m was used under PeakForce Quantitative Nanomechanical Mapping (QNM) mode. PeakForce QNM is a special kind of imaging mode of AFM based on PeakForce Tappingtechnology. In this technique each tip-sample interaction is considered to extract quantitative nanomechanical properties such as elastic modulus, adhesion, deformation, and dissipation. This technique allows mapping the above mentioned properties quantitatively and at high resolution at every pixel and at the same rate of the normal topography map. Each adhesion force image consists of 512 × 512 pixels and each pixel indicates the quantitative adhesion force extracted from the force versus separation plot in a single nanoindentation.

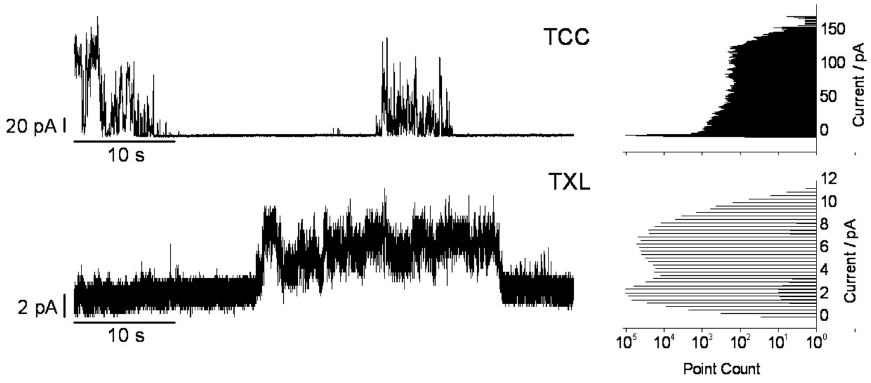

Figure S1. CDs thiocolchicoside, a colchicine derivative (TCC) and taxol (TXL) (both at 90 μM) permeabilize lipid bilayer membranes by inducing nonzero current events, toroidal pores. Here, buffer pH = 5.7, applied trans bilayer potential V = 100 mV. Both traces were filtered at 20kHz, but the lower one shows higher noise due to its presentation (current axis) at amplified scale. pA for pico ampere current. For details see refs. [2,3].

Independent Cell and Colchicine Characterization

Figure S2a presents an inverted optical microscope image, where position of the probe and distribution of cells at a large scale is clearly seen. The area of the image is (700 × 520) μm². A large scale AFM topography image of cell distribution is presented in Figure S2b. The average size of the cell is approximately 1.2 μm. The shape of all cells are not identical. This is expected because shape of cell on surface depends on the range of interaction between the cells and the surface. The shape of the cell also can be deformed due to tip-sample interaction during scanning the probe over the surface.

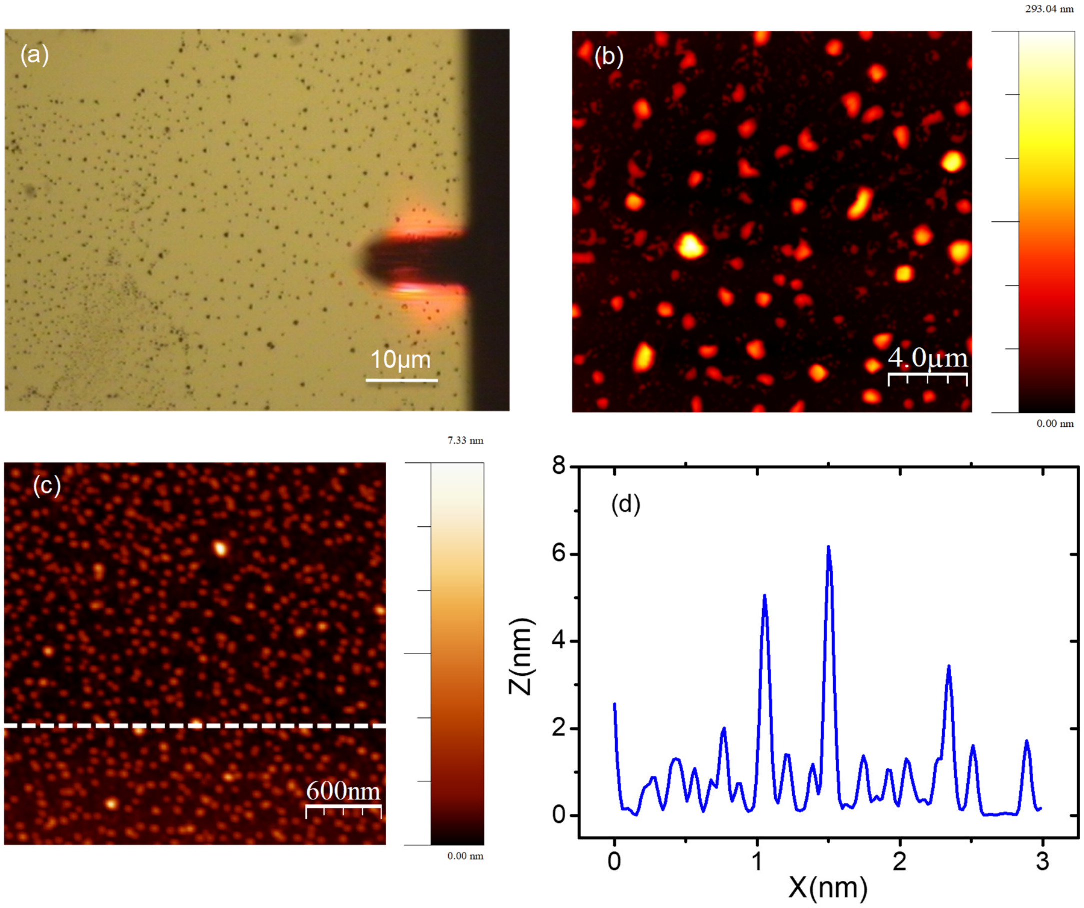

Figure S2. Cell and colchicine TM-AFM image on SiO_2 substrate: (**a**) Optical microscope image at large scale. (**b**) AFM height image of distribution of cells at a large scale. The height of the cells are presented in color scale 0 (dark fields) – 293.04 nm (light fields). (**c**) AFM height image of distribution of colchicine (1 μM) molecules. The height of the colchicine molecules are presented in color scale 0 (dark fields) – 7.33 nm (light fields). (**d**) A height profile recorded along the dashed line in Figure 1c.

The main goal of the present study is to observe the drug binding to the cell surface. In this case study, colchicine has been used as a drug. In order to observe the distribution of colchicine molecules onto the solid surface, the colchicines were also deposited onto the SiO_2 surface from their solution and AFM height image was recorded. Supplementary Figure S2c depicts the distribution of colchicine molecules on SiO_2 surface. A height profile recorded along the dashed line (Supplementary Figure S2c) is presented in Supplementary Figure S2d which shows that the height ranges approximately between 1–6.2 nm. The diameter of a single colchicine molecule obtained from its x-ray crystallographic data is approximately 1 nm (see our plot in Supplementary Figure 4). Even when taking into account that the image is widened by the convolution of the real topography with the AFM

tip [4], the height profile (Supplementary Figure 2d) indicates that we mapped a single colchicine molecule as well as a few small size clusters of colchicine molecules by AFM (Supplementary Figure S2c). Here to mention that the radius of curvature of the AFM probe used in this study was ~50 nm.

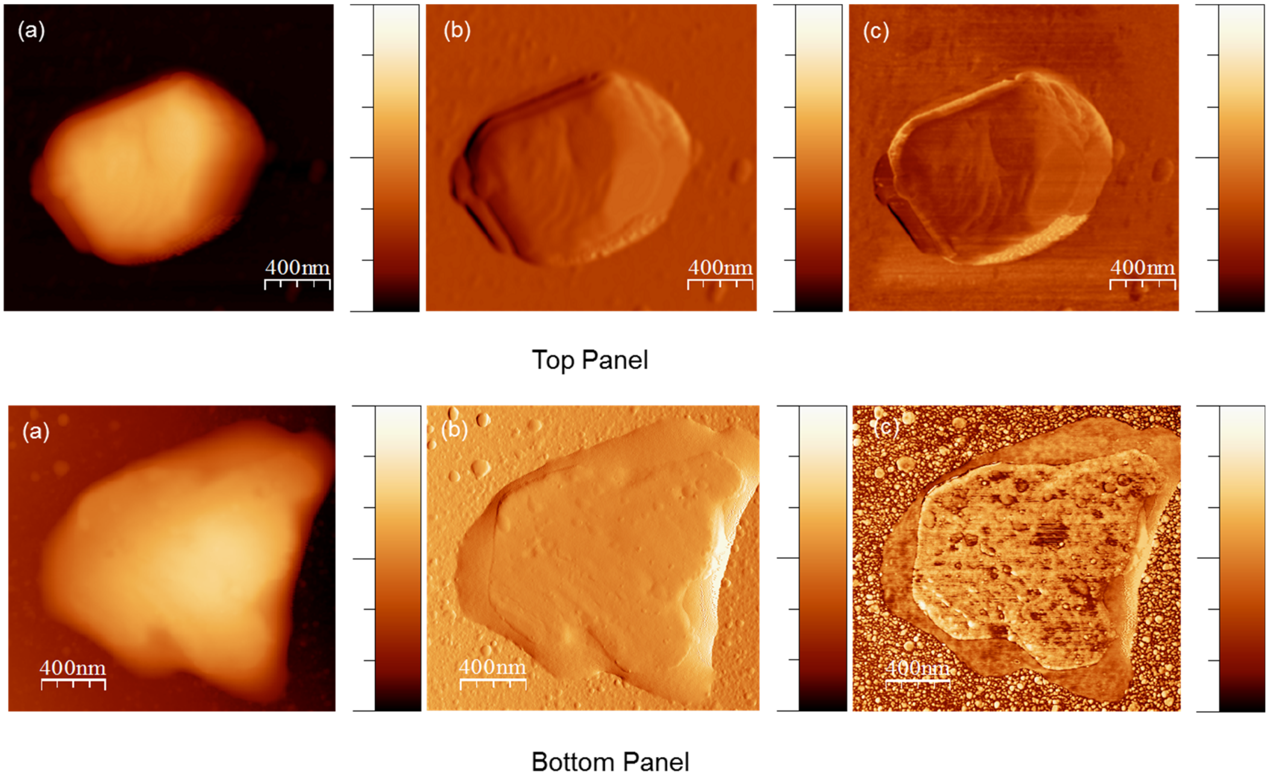

Figure S3A. TM-AFM image of a single cell on SiO_2 substrate with and without colchicine binding: Top panel represents images of a bare cell (**a**) AFM height image. The cell height is presented in color scale 0 (dark fields) – 390 nm (light fields). (**b**) AFM amplitude error image. The amplitude of a cell is represented in color scale 0 (dark fields) – 157 mV (light fields). (**c**) AFM phase image of the cell. The phase of the cell is represented in color scale 0 (dark fields) – 67 Deg (light fields); Bottom panel presents images of 1 µM colchicine treated cells. (**a**) AFM height image, the height of the cell is represented in color scale 0 (dark fields) – 319 nm (light fields). (**b**) AFM amplitude error image, the amplitude of the cell is represented in color scale 0 (dark fields) – 119 mV (light fields). (**c**) AFM phase image of the cell, the phase of the cell is represented in color scale 0 (dark fields) – 65 Deg (light fields).

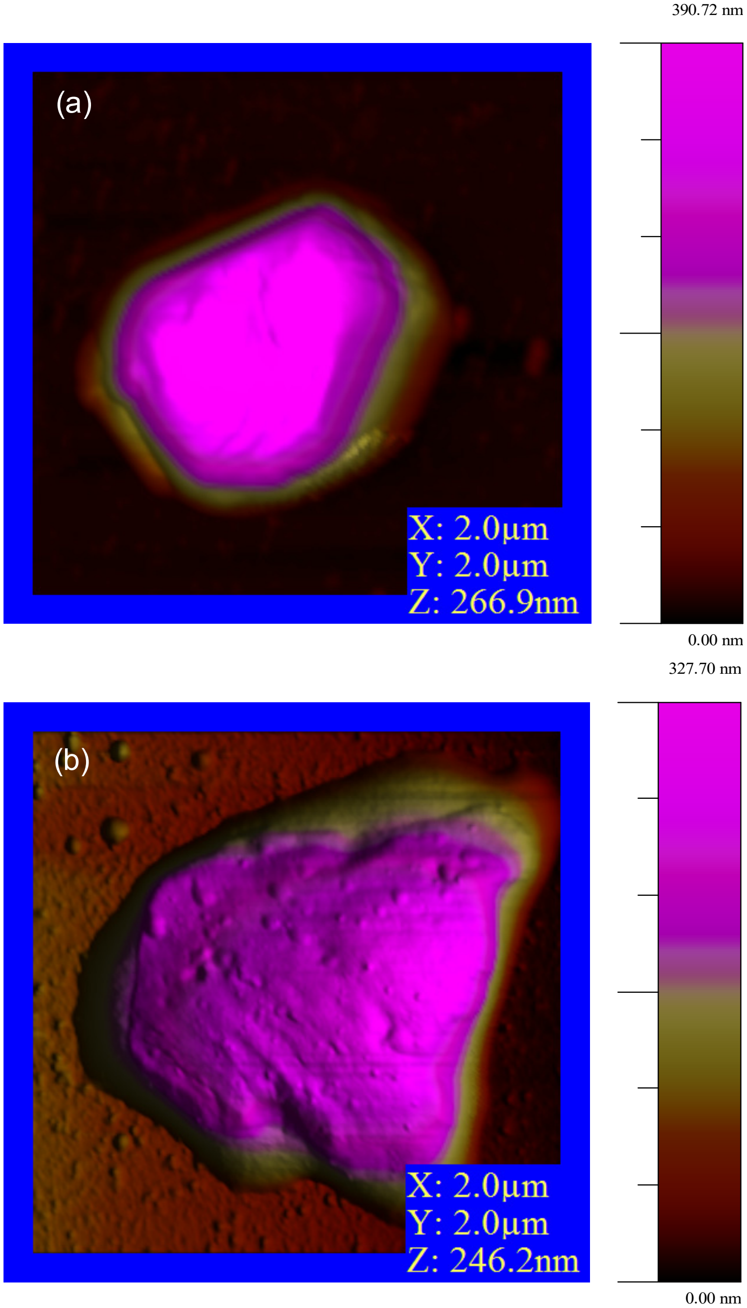

Figure S3B. Three-dimensional (3D) TM-AFM height image of a single cell in Figure 1a (top and bottom panels) with and without colchicine binding on SiO_2 substrate: (**a**) A single cell without colchicine binding. The height of the cell is presented in color scale 0 (dark fields) – 390.72 nm (light fields). (**b**) A 1 µM colchicine treated cell. The height of the cell is presented in color scale 0 (dark fields) – 327.70 nm (light fields). It is clearly seen that the cell is sitting on the liquid.

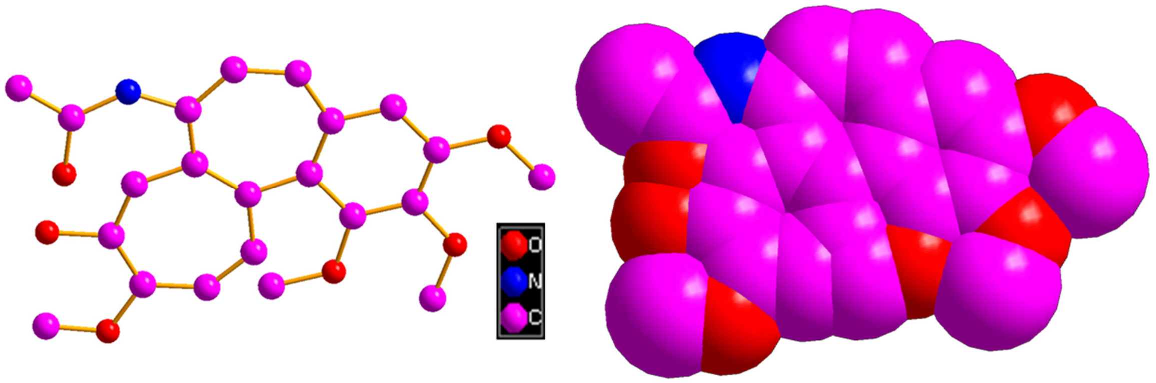

Figure S4. The diameter (d_{col}) of a single colchicine molecule is approximately, $d_{col} \approx 1$ nm. Minimum 1 nm and maximum 1.18 nm depending on the orientation have been measured using crystallography software 'Diamond'. Left: A ball-stick representation of a colchicine molecule. Right: A space-filling representation of the same molecule. Hydrogen are omitted for clarity.

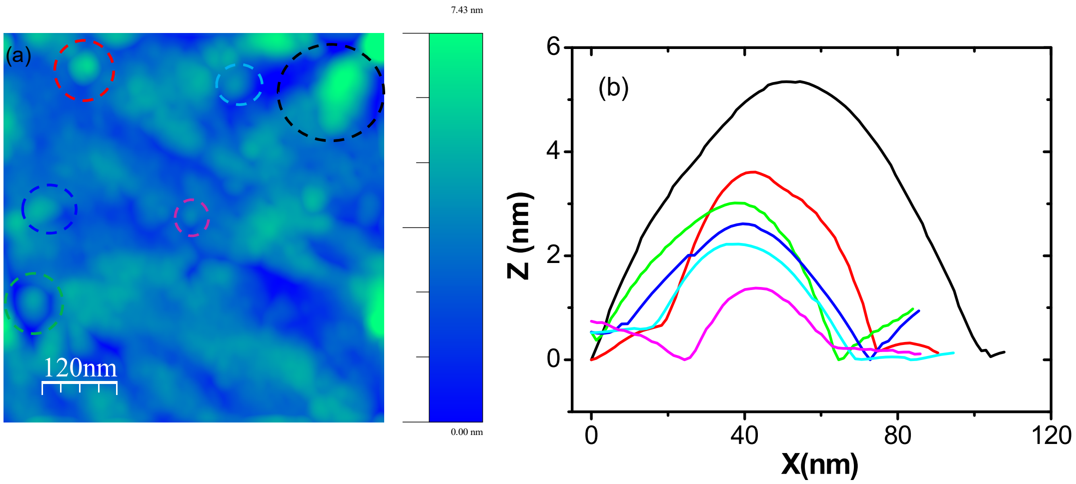

Figure S5. (**a**) A high resolution AFM height image of a 1 μM colchicine treated cell wall bound to SiO_2 substrate surface. The height of the cell wall is presented in color scale 0 (dark fields) – 7.43 nm (light fields). (**b**) A number of height profiles recorded through the center of the circles marked in Supplementary Figure S5a. Here we marked the blobs (or clusters of colchicine molecules) of different sizes by circles of various colors. A number of height profile were recorded through the centers of the circles in Supplementary Figure S5a and presented in Supplementary Figure S5b, which ranges approximately between 1.1–5.5 nm. Therefore, it can be concluded that the colchicine molecules bind to the cell membrane either in single or cluster form.

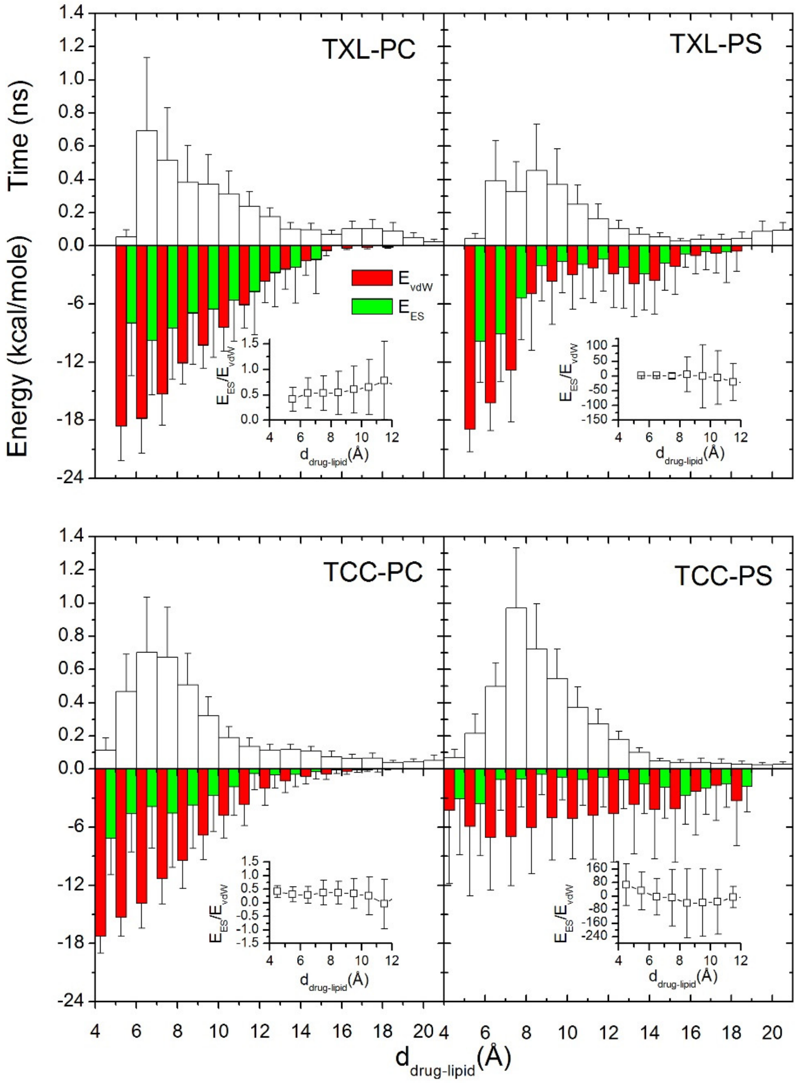

Figure S6. Here energies are detected from MD simulation on drug-lipid complexes. Simulations have been performed on two of the major cell membrane constituent lipids phosphatidylcholine (PC) and phosphatidylserine (PS) to address their interaction with cell membrane adsorbed CDs. In all four histogram plots (upper panel) of time versus $d_{drug-lipid}$ thetime durations when drug/lipid stay together (height) within a distance (width) during 6 ns simulations are presented. Lower panels show the histograms of non-bonded van der Waal's (vdW) energy (E_{vdW}) and electrostatic (ES) interactions energy (E_{ES}). To avoid color conflict E_{vdW} and E_{ES} are shown to occupy half-half widths though each half represents the whole width of the corresponding histogram. For details see ref. [3].

References

1. Shiba, M.; Watanabe, E.; Sasakawa, S.; Ikeda, Y. Effects of taxol and colchicine on platelet membrane properties. *Thromb. Res.* **1988**, *52*, 313–323, doi:10.1016/0049-3848(88)90072-2.
2. Ashrafuzzaman, M.; Duszyk, M.; Tuszynski, J. Chemotherapy drug molecules thiocochicoside and taxol permeabilize lipid bilayer membranes by forming ion channels. *J. Phys. Conf. Ser.* **2011**, *329*, 012029.
3. Ashrafuzzaman, M.; Tseng, C.-Y.; Duszyk, M.; Tuszynski, J.A. Chemotherapy Drugs form Ion Pores in Membranes Due to Physical Interactions with Lipids. *Chem. Biol. Drug Des.* **2012**, *80*, 992–1002, doi:10.1111/cbdd.12060.
4. Rosenman, S.J.; Ganji, A.A.; Gallatin, W.M. Contact dependent redistribution of cell surface adhesion and activation molecules reorganization. *FASEB J.* **1991**, *5*, 1603.
